# The future of recombinant host defense peptides

**DOI:** 10.1186/s12934-022-01991-2

**Published:** 2022-12-21

**Authors:** Ramon Roca-Pinilla, Leszek Lisowski, Anna Arís, Elena Garcia-Fruitós

**Affiliations:** 1 Translational Vectorology Research Unit, Faculty of Medicine and Health, Children’s Medical Research Institute, The University of Sydney, Westmead, NSW 2145 Australia; 2 Laboratory of Molecular Oncology and Innovative Therapies, Military Institute of Medicine, Warsaw, Poland; 3 Department of Ruminant Production, Institut de Recerca i Tecnologia Agroalimentàries IRTA, 08140 Caldes de Montbui, Spain

**Keywords:** Host defense peptides, Antimicrobial proteins, Antimicrobial resistance, Inclusion bodies, Recombinant production

## Abstract

The antimicrobial resistance crisis calls for the discovery and production of new antimicrobials. Host defense peptides (HDPs) are small proteins with potent antibacterial and immunomodulatory activities that are attractive for translational applications, with several already under clinical trials. Traditionally, antimicrobial peptides have been produced by chemical synthesis, which is expensive and requires the use of toxic reagents, hindering the large-scale development of HDPs. Alternatively, HDPs can be produced recombinantly to overcome these limitations. Their antimicrobial nature, however, can make them toxic to the hosts of recombinant production. In this review we explore the different strategies that are used to fine-tune their activities, bioengineer them, and optimize the recombinant production of HDPs in various cell factories.

## Background

In 2019 alone, 1.27 million people died globally due to antimicrobial-resistant bacteria (ARB) [1]. At the current rate of resistance development, 10 million people will die by 2050 due to our inability to treat infections [1]. There is, therefore, a severe need to find suitable alternatives as effective as conventional antibiotics or that can be used in a combinatorial treatment [2].

One potential alternative is the use of host defense peptides (HDPs), which are a diverse and well-studied class of bioactive peptides (AMPs) that all multicellular organisms produce as a defense mechanism against pathogenic microbes [3, 4]. HDPs were discovered in the 1980s thanks to the keen eye of researchers that could not explain what they observed with their current understanding of immunity. For instance, *Cecropia* moth pupa that lacked antibodies or lymphocytes were still able to resist bacterial infections thanks to the action of cecropin [5]. Another example is the potent antimicrobial activity of rabbit neutrophils due to defensins [6] and the skin wound-healing abilities of an African clawed frog, thanks to secreted magainins [7]. Since then, the field of HDPs exploded and today there are more than 3000 known peptide sequences that come from all domains of life, including HDPs [8].

The characteristics that usually define HDPs are their short amino acidic sequences (between 12 and 50 amino acids) [4], a net positive charge [5], a certain degree of hydrophobicity [9] and a wide range of broad-spectrum biological activities [10]. Among these activities, HDPs have microbicidal (effective against bacteria, virus, and fungi) [11–13], antibiofilm [14, 15], and immunomodulatory activities [16–18]. Interestingly, it might be difficult for microorganisms to develop resistance against HDPs because of their multiple modes of action, which may ultimately lead to microbial death [3].

**Fig. 1:**
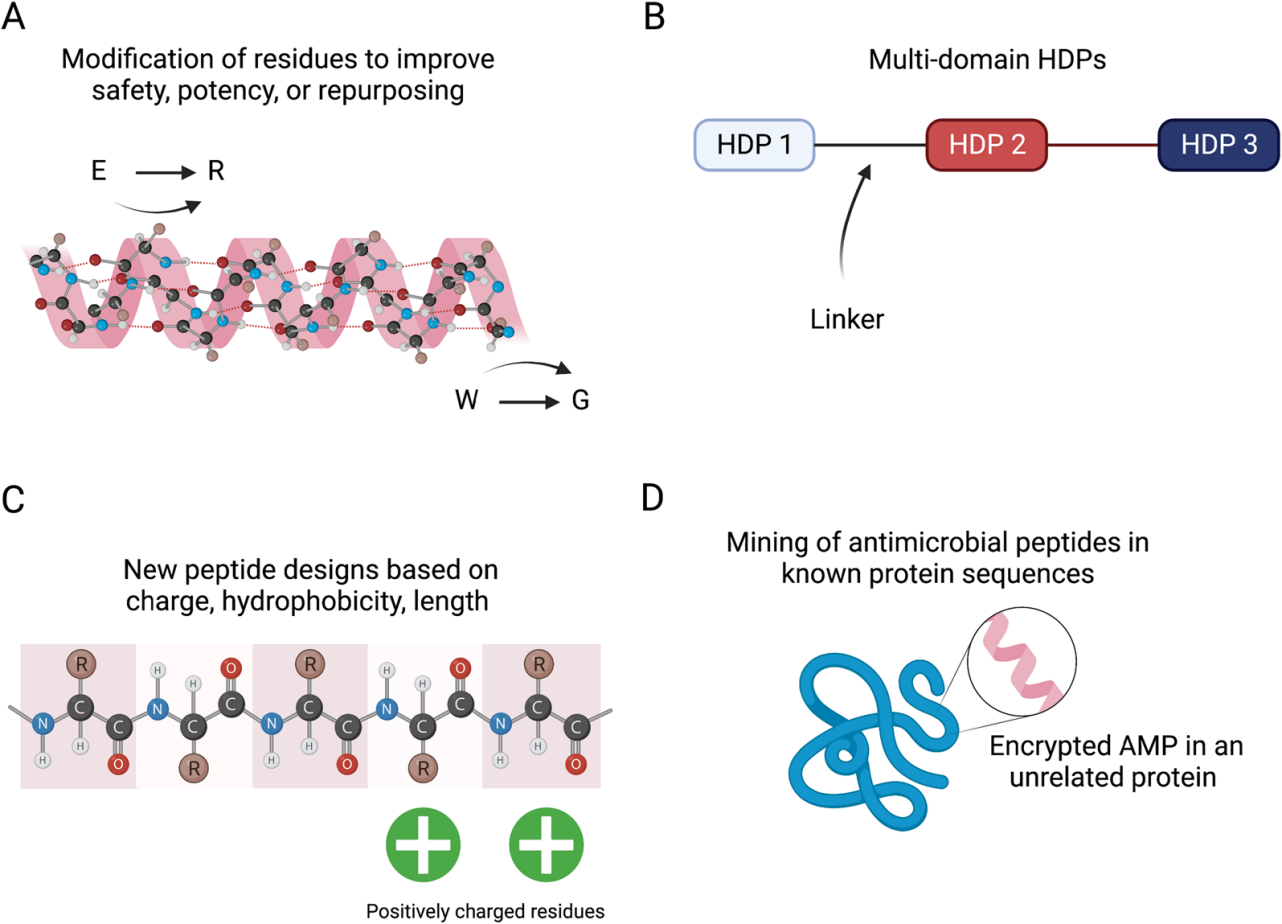
Schematic figure showing several strategies that can be used to engineer antimicrobial peptides. **A** Rational modification of peptides to improve some of their features; **B** multidomain proteins based on the combination of different HDPs; **C** design new peptides based on their known properties; or **D** find encrypted peptides “buried” in known protein sequences

Their well-known characteristics make them amenable to engineering [19–21] (Fig. 1A, B), peptide repurposing, such as engineered venoms that can be modified to become non-toxic HDPs [22] (Fig. 1A), development of multidomain proteins based on the combination of different HDPs (Fig. 1B), de novo designs [9, 23] (Fig. 1C), or aid in the discovery of hidden peptides within larger protein structures [24] (Fig. 1D). All these new technologies yield an almost unlimited potential to modify known sequences or discover new peptides and modes of action.

There are multiple ways to classify HDPs, considering their secondary structure or common ancestry, for example. In vertebrates, there are two major families of HDPs: cathelicidins and defensins [10]. The latter have a common β-sheet core stabilized by three disulphide bridges. Depending on how the cysteine residues link together, defensins are classified into α-, β-, and θ-defensins [10]. Instead, over one third of the cathelicidins are α-helical. Cathelicidins are produced as prepropeptides that need to be secreted and then cleaved by serine proteases [25]. However, there are other families, such as histatins, which are histidine rich HDPs from mammals’ saliva (Fig. 1D) [10, 24, 26].

Although some peptide-based antimicrobials are in advanced clinical trials, none of them has been granted regulatory approval [27]. There are many reasons, beyond the scope of this review, as to why that is the case, including potential toxicity, low stability, half-life, and a high production cost.

Together with the need to be safe and effective, one of the big questions that remains is how to produce them in large quantities in a sustainable way and with a competitive price. Besides, some strategies including chemical modifications and delivery vehicles are being studied to improve the properties of these peptides [28].

**Fig. 2:**
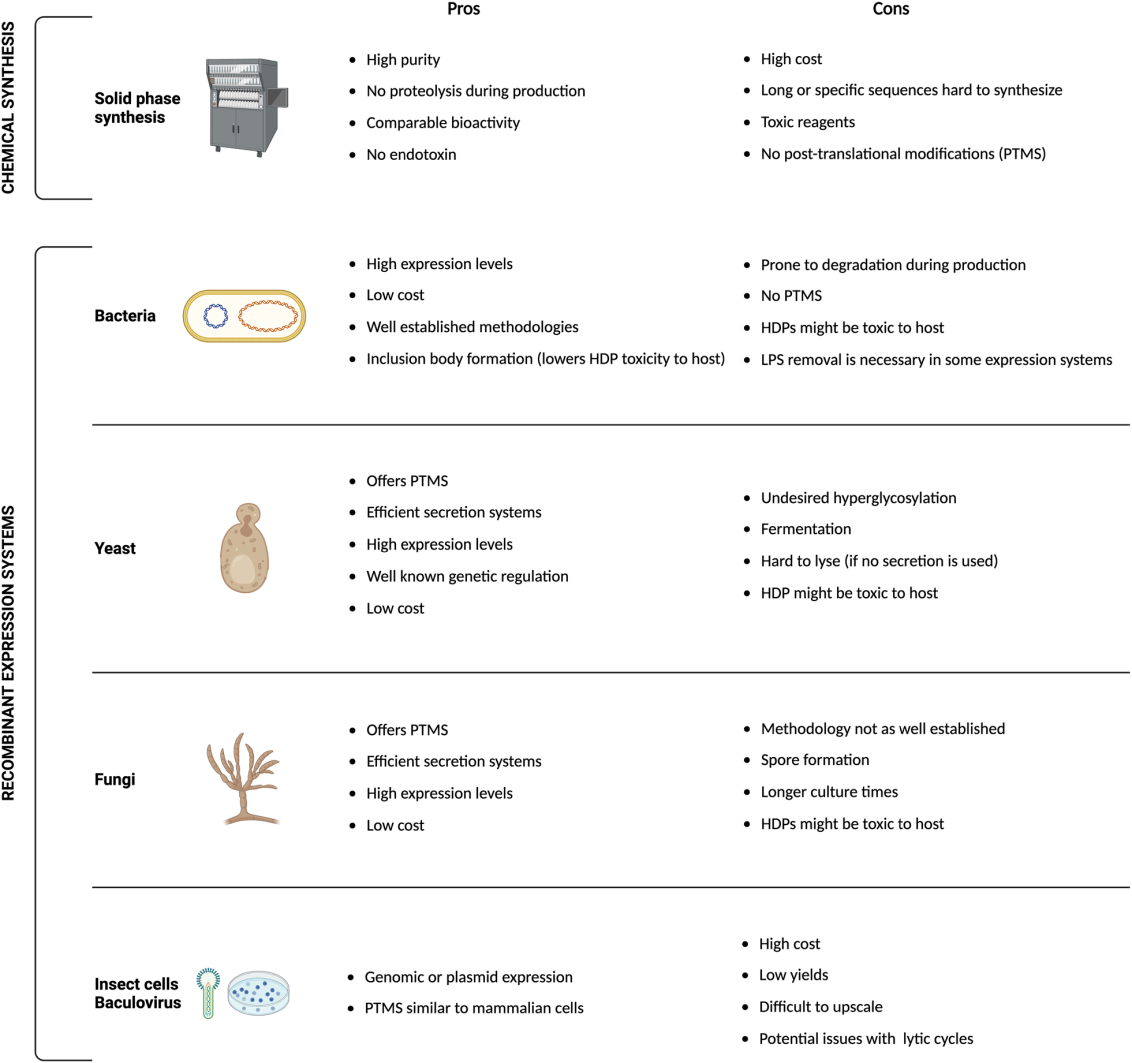
Advantages and disadvantages of different recombinant expressions systems to produce HDPs [52, 108–110]. Only the most utilized systems are shown, as there are very few instances where mammalian cells have been used for recombinant HDP production, as it is probably too expensive and unnecessary in many cases. The bulk of the work with plant systems is based on transgenic plants to enhance their properties with genetic modifications, but not necessarily recombinant HDP production per se

The first HDPs were isolated and purified from their natural sources, but this process is difficult and time-consuming, and results in low yields, making it difficult to scale up (Fig. 2). Most of the studies to date use chemically synthesized HDPs [29], and, by automated solid-phase peptide synthesis (SPPS) [29]. This strategy involves the repetitive binding of different amino acids to obtain the desired peptide sequence, which is bound to a resin support. Once the sequence is obtained, the peptide can be retrieved from the solid support with high purity via a cleavable linker.

Yet, in addition to the high production costs, one of the main problems of chemical synthesis is its environmental impact, due to the excessive use of organic solvents during the process [30]. Thus, it does not suit the needs of large-scale production [31]. Besides, chemical synthesis can be tricky for longer peptide sequences of more than 35 amino acid residues [32]. In this context, our goal in this review is to discuss the feasibility of recombinant HDP production as an alternative to chemical synthesis, considering several of the microbial cell factories that have been used so far, as well as various protein forms and strategies that lead to success in recombinant HDP production endeavors.

## Cell factories for recombinant HDP production

As an alternative to chemical synthesis, technologies based on recombinant DNA have been explored for the biosynthesis of HDPs. The recombinant production of these peptides offers a more flexible, sustainable, scalable, and cost-effective production [31]. Many organisms can be used as hosts for recombinant HDP production, including plants, insect cells, mammalian cells, yeast, and bacterial cells [33]. However, choosing the optimal expression organism is critical to ensure proper protein yields, biological function, and final cost (Fig. 2).

### Recombinant production in bacteria

The most extensively used host for recombinant expression of proteins and peptides are bacteria, as they are easy to manipulate, grow fast and use inexpensive media. Nevertheless, bacteria, have a limited ability to make disulphide-bonds, glycosylation, and other post translational modifications (PTMS) [34–36]. These limitations, however, might not be critical for HDP recombinant production [37]. In the absence of a strict need for PTMS, the heterologous expression in bacteria is a reasonable approach for their production with sufficient conformational and functional quality.

As it occurs for many other recombinant proteins, the most utilized bacterium for HDP production is *E. coli* (Table 1), since it has been widely studied as a recombinant host, with an extensive knowledge of its genetics, biochemistry, and physiology [38]. There are well-established protocols and a large catalogue of expression vectors, and besides, it grows fast. In addition, some HDPs have been successfully expressed in *Lactococcus lactis* (Table 1) and *Bacillus subtilis.* Although there are only few examples of HDPs produced in these LPS-free bacteria, they are appealing candidates considering their status as generally regarded as safe (GRAS). Most HDPs expressed in bacteria are produced with inducible expression systems yielding quantities that range between 2 and 600 mg/L [39].

**Table 1:** Recombinant expression of HDPs and the different cell hosts used to express them

HDP Type	Family	Host	Protein form^a^	Fusion tag	References
Apidaecin	Insect AMP	*L. lactis* subsp. cremoris NZ9000	Secreted	–	[111]
Apidaecin	Insect AMP	*Pichia pastoris*	Secreted	Human serum albumin (HSA)	[43]
Big defensins	Molusc big defensin	*E. coli* BL21(DE3)	Solubilized	–	[112]
Bovine lingual antimicrobial peptide (LAP)	Bovine defensin	*E. coli* BL21(DE3) *E. coli* Origami B (DE3)	Soluble Solubilized	eGFP	[37]
Buforin II	Histone H2A-derived AMP	*E. coli* BL21 (DE3)	Solubilized	Cystein-rich acidic peptide (CAP)	[92]
Cathelicidin-BF (CBF)	Cathelicidin	*B. subtilis* WB800 N	Secreted	Intein SUMO	[95, 157]
CcDef2gene	Insect defensin	*E. coli* BL21(DE3)	Solubilized	3xCcDef2 Casette	[113]
Cecropin	Cathelicidin	*P. pastoris*	Soluble	Oleosin	[114]
ChMAP-28, mini-ChBac7.5Nα, and mini-ChBac7.5Nα	Goat cathelicidins	*E. coli* BL21(DE3)	Solubilized	Trx	[115]
CRAMP	Cathelicidin	*E. coli* BL21(DE3)	Soluble	SUMO	[41]
Cryptidin-2	Defensin	*E. coli* Rosetta-gami B (DE3)	Soluble	Trx	[64]
E5 and E6	Bovine bactenecin derivative	*E. coli* BL21(DE3)	Soluble	SUMO	[41]
Egyptian maize defensin (MzDef)	Plant defensin	*E. coli* BL21(DE3)	Soluble	GST	[116]
FaAMP	Fungal defensin	*E. coli* BL21 (DE3)	Soluble	eGFP	[117]
fBD	Flounder defensin	*E. coli* BL21(DE3)	Soluble	–	[118]
Fowlicidin-1	Chicken cathelicidin	*E. coli* BL21 (DE3)	Soluble	Calmodulin	[119]
Fungal defensin-like peptide (DLP)	Fungal defensin	*P. pastoris*	Secreted	–	[120]
GL13K	Encrypted peptide^a^	*E. coli* BLR	Soluble	Elastin-like recombinamers	[121]
Gloverin	Insect antibacterial protein	*Drosophila melanogaster S2*	Secreted	–	[52]
Human neutrophil peptide 1 (HNP1)	Human defensin	*P. pastoris*	Secreted	Polyhedrin-eGFP	[45]
Human neutrophil peptide-1 (HNP-1)	Human defensin	*E. coli* strain XPX-1	Soluble	–	[122]
Human α-defensin 5 (HD5)	Human defensin	*E. coli* BL21(DE3) *E. coli* Origami B (DE3)	Solubilized	eGFP Trx	[20, 21, 37]
Human α-defensin 5 (HD5)	Human defensin	*P. pastoris*	Secreted	Alpha-factor	[123]
Human β-defensin 1 (HBD1)	Human defensin	*E. coli* AD202	Solubilized Soluble	C-terminal fragment of light meromyosin (LMM) Trx	[124, 125]
Human β-defensin 1 (HBD1)	Human defensin	*S. cerevisiae* AH22	Secreted	–	[126]
Human β-defensin 118	Human defensin	*E, coli* Rosetta (DE3)	Soluble	–	[127]
Human β-defensin 2 (HBD2)	Human defensin	*E. coli* BL21(DE3)	Soluble Solubilized	Trx Keto-steroid isomerase (KSI) Glutathione-*S*-transferase (GST)	[125, 128–131]
Human β-defensin 3 (HBD3)	Human defensin	*E. coli* BL21(DE3)	Soluble	Trx Calmodulin	[119, 132]
Human β-defensin 4 (HBD4)	Human defensin	*E. coli* BL21(DE3)	Soluble	Trx	[133]
Human β-defensin 6 (HD6)	Human defensin	*E. coli* Origami (DE3) pLys	Soluble	Trx	[134, 135]
Human β-defensin DEFB136	Human defensin	*E. coli* BL21(DE3)	Soluble	Intein-chitin binding domain (CBD)	[136]
Hybrid peptide Cecropin AD	Cecropin	*B. subtilis* WB800N	Secreted	Small ubiquitin modifier (SUMO)	[137]
IDR-1	Innate defense regulator (IDR)	*E. coli* BL21(DE3)	Soluble	SUMO	[41]
Indolicidin	Cathelicidin	*E. coli* BL21 (DE3)	Soluble	Calmodulin	[119]
Insect defensin A	Defensin	*S. cerevisiae*	Secreted	Yeast pheromone mating factor α (MFα)	[138]
Lactoferrampin B	Fragment of lactoferrin	*E. coli* BL21 (DE3)	Soluble	Calmodulin	[119]
LL-37	Cathelicidin	*E. coli* BL21 (DE3)	Soluble	SmbP Silk SUMO	[41, 139]
LsGRP1c	Glycine Rich Protein from	*E. coli* BL21 *E. coli* C41 (DE3) *E. coli* C43 (DE3) *E. coli* C41 (DE3) pLysS *E. coli* C43 (DE3) pLysS	Soluble	SUMO	[140]
LvCrustinVII	Crustacean AMP	*E. coli* BL21(DE3)	Solubilized	–	[141]
Magainin II F5W	Cathelicidin	*E. coli* BL21 (DE3)	Soluble	Calmodulin	[119]
Magainin II-cecropin B chimera	Magainin/cathelicidin hybrid	*Cordyceps militaris*	Secreted	Chimeric protein^b^	[50]
Melittin	–	*E. coli* BL21 (DE3)	Soluble	eGFP Calmodulin	[117, 119]
MIP-3α_51-70_	Chemokine fragment	*E. coli* BL21 (DE3)	Soluble	Calmodulin	[119]
MX226	Indolicidin derivative	*E. coli* BL21(DE3)	Soluble	SUMO	[41]
OrR214 and OrR935	Rice AMPs	*B. subtilis* SCK	Secreted	–	[142]
pBD-2-cecropin P1 chimera	Defensin/cathelicidin hybrid	*B. subtilis*	Secreted	Chimeric protein^b^	[143]
Peptide P2	Designed peptide	*E. coli* NM522	Solubilized	Bovine prochymosin	[144]
Pexiganan	Magainin analogue	*E. coli* BL21 (DE3)	Soluble	DAMP4	[145]
Pexiganan-honeybee silk chimera (modified magainin-2)	Magainin analogue/silk-fibre hybrid	*E. coli* Rosetta 2 (DE3)	Solubilized	Silk	[146]
Plectasin	Fungal defensin	*B. subtilis* WB800N	Secreted	SUMO	[147]
Plectasin	Fungal defensin	*P. pastoris*	Secreted	4xPlectasin casette	[46]
Porcine β-defensin 2 (pBD-2)	Porcine defensin	*E. coli* BL21(DE3)	Soluble	–	[141, 148]
PsDef5.1	Fungal defensin	*E. coli* BL21(DE3) CodonPlusRIL Rosetta-gami 2(DE3)	Soluble	Thioredoxin (Trx)	[149]
Puroindoline A	–	*E. coli* BL21 (DE3)	Soluble	Calmodulin	[119]
r(P)ApoBL, r(P)ApoBs^a^	Encrypted peptide^a^	*E. coli* BL21 (DE3)	Solubilized	Onconase	[150]
rAvBD1-2–6–13	Chicken defensin	*L. lactis* NZ3900	Soluble	–	[151]
Scorpine	Defensin	*Anopheles gambie*	Secreted	–	[51]
Sericin-cecropin	Silk-fibre/cathelicidin hybrid	*E. coli* BL21 (DE3) *E. coli* Rosetta (DE3)	Soluble	Silk	[152]
*Sesvania javanica defensin* (Javanicin)	Defensin	*E. coli* Origami 2 (DE3)	Soluble	Intein-CBD	[153]
SMAP	Cathelicidin	*E. coli* BL21 (DE3)	Soluble	eGFP	[117]
Snakin-1 (StSN1)	Plant AMP	*Spodoptera frugiperda* (Baculovirus-infected insect cells)	Secreted	–	[154]
T9W	Variant of pig myeloid antimicrobial peptide-36	*B. subtilis* WB800N	Secreted	SUMO	[155]
Thanatin	Insect AMP	Human Embryonic Kidney (HEK)293	Secreted	–	[156]
Tilapia piscidin	Piscidin	*P. pastoris*	Soluble	–	[44]
Tritrpticin	Cathelicidin	*E. coli* BL21 (DE3)	Soluble	Calmodulin	[119]

Histidine tags were not considered to be fusion tags that help to express HDPs

Soluble: soluble protein produced in the cytoplasm; solubilized: protein solubilize from IBs; secreted: soluble protein secreted to the media

^a^Denotes peptide fragment obtained from larger, non-antimicrobial proteins

^b^We considered chimeric proteins those that do need cleaving of the tag as they add new and desired bioactivities

### Recombinant production in yeasts

Sometimes, the production of cysteine rich cationic HDPs fails in bacterial systems such as *E. coli*, due to an inefficient formation of disulphide bridges that leads to improper folding and lack of bioactivity [40]. Another concern when expressing HDPs in bacteria is their natural lethality towards the host [41]. Therefore, switching to eukaryotic cells can be a suitable alternative to overcome this challenge, allowing for the heterologous expression of HDPs. Yeasts are one of the simplest eukaryotic organisms [42] and offer a good compromise between the higher complexities of eukaryotic cells and the relatively simple and inexpensive recombinant production of prokaryotic systems (Fig. 2). Besides, they grow faster than typical mammalian recombinant hosts such as Chinese Hamster Ovary (CHO) or Human Embryonic Kidney (HEK293) cells. And yeasts are free from endotoxins and harmful human viruses and can secrete large amounts of heterologous recombinant proteins with little host cell protein secretion, which can simplify downstream purification (Fig. 2).

Exploiting all of these, a group from MIT [43] expressed apidaecin, an insect HDP, fused to human serum albumin (HSA) in the yeast *Pichia pastoris*. They obtained yields of more than 700 mg/L with no cell lysis and no debris removal steps required. However, because it is a fusion construct, downstream cleaving of the fusion tag was necessary. Fish [44], human [45] and fungal [46] peptides have also successfully been produced recombinantly in *P. pastoris* (Table 1), showing evidence that it can be a useful alternative to the most used expression system for recombinant production (*E. coli*). *Saccharmoyces cerevisiae* is another yeast-based system that has been used for the expression of HDPs [47, 48], albeit less than *P. pastoris*. The yields (mg/L) for HDPs expressed in yeast found in the relevant literature are highly variable [39], between less than 0.1 to up to 831 mg/L, suggesting the need to fine tune the expression system for each of the peptides.

### Recombinant production in fungi

The use of filamentous fungi for HDP production is still in its infancy. However, interest in their use is growing due to their successful application in the recombinant production of non-antimicrobial proteins, their ability to perform PTMS, scale-up ability, and inexpensiveness of culture. The fungal defensin plectasin, developed for the treatment of Gram-positive bacterial infections is produced recombinantly using *Aspergillus oryzae* as a high efficiency recombinant protein expression system, produced as a secreted product (Table 1) [49]. Another example is the recombinant production of a hybrid HDP of magainin II-cecropin B, successfully expressed in *Cordyceps militaris* with a yield of 3.86 mg/g of mycelium (Table 1) [50].

### Recombinant production in insect cells

The first isolated HDP was cecropin (1980), an insect HDP [5]. Insect cell-based systems prove to be very valuable for the recombinant expression of HDPs. In general, the most prevalent systems are based on *Drosophila melanogaster* cell lines, although there is some work in mosquito and moth cells [51]. Two main approaches are used to express HDPs in insect systems, either by using a Baculovirus gene expression system [35], or by stably expressing a HDP by means of genome integration of the HDP-coding gene.

Even though they are more expensive than yeast and prokaryotic-based systems, they offer PTMS such as glycosylation that might aid the proper structure and bioactivity of HDPs [52], although it is not clear if PTMS are necessary in all instances, or at all, suggesting that their need (or their lack of) might require to be studied on a case-by-case basis. However, when the Baculovirus system is used, the lytic cycle of the virus can cause large amounts of recombinant peptide loss due to degradation [53]. Yields of HDPs produced in insect systems are less well documented than for yeast or bacterial systems, and range between 6 and 25 mg/L [39].

## Forms of recombinant HDPs

In general, recombinant HDPs have been produced in 3 main forms (Table 1): soluble protein (secreted or intracellular), inclusion bodies (IBs) or as encapsulated soluble protein.

### Soluble recombinant production

Many HDPs have been successfully obtained through recombinant production in different expression systems in a soluble form (Table 1) [54, 55]. In *E. coli* and *L. lactis*, HDPs are typically produced intracellularly and purified after cell disruption (Table 1). In contrast, when *B. subtilis*, yeast or fungi are used, the sequences of the proteins of interest are, mostly, designed to be secreted to the growth media (Table 1). However, in many other cases, especially when using bacterial expression systems, HDPs aggregate forming IBs, being necessary to solubilize these protein aggregates (Table 1), as detailed in “IBs as a source of soluble HDPs” section.

Besides aggregation, another issue of recombinant bacterial expression of HDPs is their potential lethality to the recombinant host due to their antibacterial nature [56, 57]. In addition, they are highly susceptible to proteolysis due to their small size and positive charge. To overcome all these problems, the most common strategy is to produce soluble HDPs with a fusion partner or to produce them as multidomain proteins, as described in detail in “Strategies to optimize HDP production” section.

#### Encapsulated soluble HDPs

Some groups have also been working on the development of HDP delivery systems to have a time-controlled release to improve bioavailability and to minimize toxicity and proteolytic degradation and, in consequence, increase stability, when administered in vivo [58]*.* Most of these studies, however, have been done using synthetic HDPs. For example, different HDPs have been encapsulated using polymeric lactic-co-glycolic acid (PLGA) nanoparticles, showing that the encapsulated peptide kept the antimicrobial activity and did not increase toxicity when compared to the naked peptide [59, 60]. Other studies using PLGA microspheres decorated with *N*-acetyl cysteine (NAC) for pulmonary drug delivery have shown to have a good potential both in vitro and in vivo [61]. Synthetic HDPs have been also nanoencapsulated in lipid-based nanoparticles. A peptide from the cathelicidin family was encapsulated in liposomes and the authors proved that the peptide kept its antimicrobial activity while showing a sustained release and a reduction in pro-inflammatory cytokines release when compared to the non-encapsulated peptide [62]. Another approach that has been studied is the use of Polyethylene glycol (PEG)-stabilized lipodisks to protect cationic peptides [63]. There is also a recent study that evaluated the encapsulated form of recombinant HDPs. Kaur and coauthors analyzed the PEGylated form of mouse alpha-defensin cryptdin-2 [64] and they observed a two-fold decrease in the antimicrobial activity when cryptidin-2 is conjugated to PEG. This effect could be attributed to a masking effect of PEG, and further studies are needed to evaluate the impact of PEGylation size and site, as previously described [64]. Going a step further, Drayton and coworkers have designed an enzyme-cleavable HDP-PEG system for the delivery of active HDPs, which is based on the release of the antimicrobial peptide at the site of infection after cleavage by a host enzyme [65]. Thus, although much remains to be done, the results obtained so far are promising when it comes to increase peptide stability and decrease some of the adverse effects.

### Antimicrobial inclusion bodies

IBs are protein nanoparticles or aggregates whose formation has been widely described in *E. coli* [56, 57], but also in other microbial expression systems such as lactic acid bacteria [66–70] and yeast [70, 71]. These aggregates can be easily purified [72] and offer interesting features not available in a soluble form. IBs are an active biomaterial that has already been explored in several applications such as cancer [73], biocatalysis [74], tissue regeneration [75] and immunostimulation [76], as they are highly stable protein nanoparticles with slow-release properties [56, 57, 77]. Recently, two studies have proven that HDP-based IBs are biologically active against different pathogenic bacteria [20, 37]. In the first study, López-Cano et al. showed that human α-defensin 5 (HD5) and lingual antimicrobial peptide (LAP) IBs are highly active against MRSA and *Pseudomonas aeruginosa,* with antimicrobial activities comparable to the soluble counterpart [37]. The second study showed the antibiofilm properties of IB-decorated surfaces against a carbapenem resistant *Klebsiella pneumoniae* [20], adding to the evidence that antimicrobial IBs can be effectively used against AMR bacteria.

To maintain antimicrobial activity, constant administration of an antimicrobial that has a short half-life is required, as concentrations under minimum inhibitory concentrations (MIC) will probably happen during treatment, further increasing the appearance of AMRs. This is why a slow-release profile seems to be vital to maintain constant antimicrobial levels for long periods, to get an optimal therapeutic benefit, where HDP-based IBs are promising protein format for antimicrobial applications [37]. Therefore, antimicrobial IBs can be used as nanopills that display antibacterial activity against different AMR Gram-positive and Gram-negative strains. These HDP-releasing nanopills can also be used to decorate plastic surfaces to avoid biofilm formation by bacteria [20]. In addition, the IB format per se can be antimicrobial, as one study found, where non-antimicrobial proteins such as GFP and IFN-γ, when presented as IBs, achieved a significant reduction in bacterial loads [13].

#### IBs as a source of soluble HDPs

**Fig. 3:**
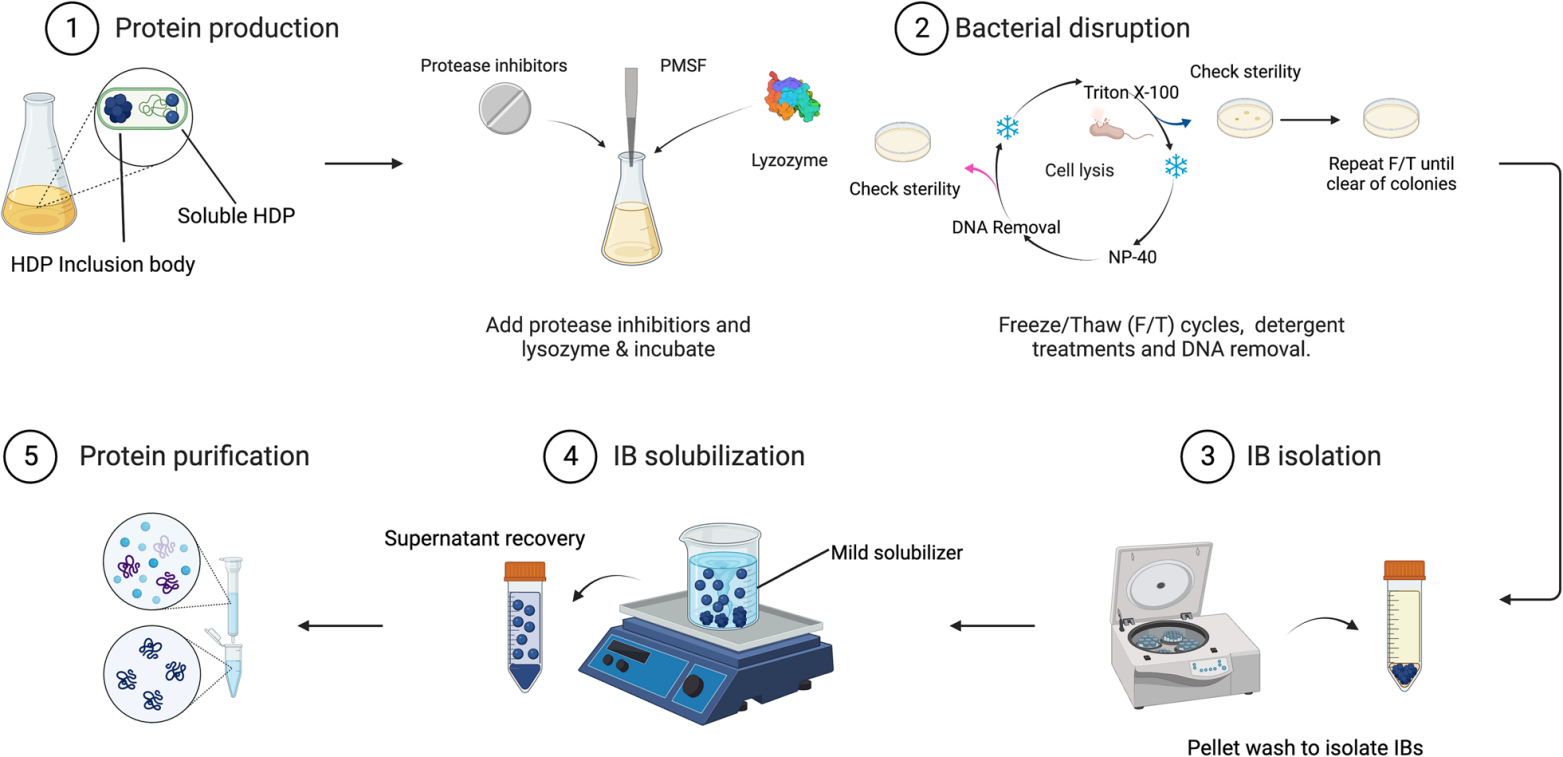
Mild solubilization of HDP produced as IB in prokaryotic systems such as *E. coli* [20]

IBs can also be used as an alternative source to obtain soluble HDPs when recombinant proteins aggregate (Fig. 3). This is useful when the isolated soluble version of the HDP is required for specific applications, but most of the protein of interest forms aggregates. It is especially relevant when HDPs are produced in *E. coli*, since in many cases it is necessary to recover the protein of interest from the aggregated fraction (Table 1). A high percentage (around 34%) of the recombinant HDPs produced in *E. coli* are extracted from IBs (Table 1).

In general terms, high concentrations of denaturing agents such as 6M GdnHCl or 8M urea are used to extract soluble protein from IBs. However, alternative protocols have been developed in the last years [78]. Since it has been widely proven that proteins embedded in these nanoparticles can still be functional, the soluble form of different proteins has also been extracted under mild, non-denaturing conditions [20, 66, 78–80]. Unlike denaturing protocols, this last strategy allows to obtain soluble protein from IBs without the need to use unfolding and refolding processes. Among these articles, some of them have already successfully tried this approach using IBs formed by antimicrobial proteins [20, 21, 37]. Indeed, solubilized IB proteins show antimicrobial activity against *E. coli* in a dose-dependent fashion against carbapenem-resistant *K. pneumoniae* embedded in biofilms [20]. However, it is important to point out that the selection of an optimal mild solubilizer is especially relevant when antimicrobial proteins are purified because recently it has been reported that they can impair the antimicrobial activity [81].

## Strategies to optimize HDP production

### Fusion tags

**Fig. 4:**
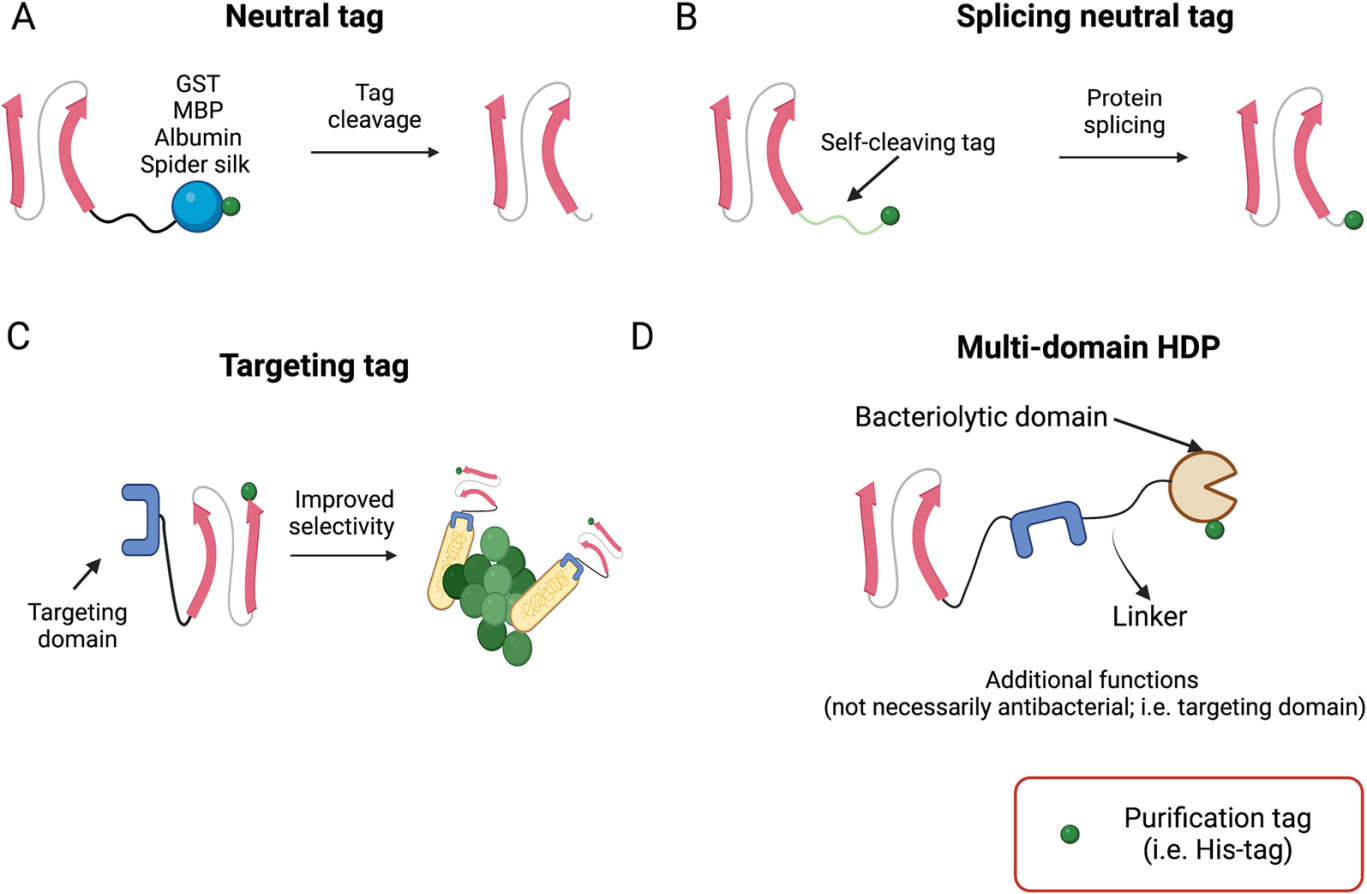
Different strategies allow the successful production of HDPs, which might otherwise be refractory to recombinant production. **A** A fusion partner that mimics the peptide precursor structure, but that must be removed downstream, **B** self-cleavable tags such as inteins, **C** specificity-targeting tags and **D** a multi-domain HDP combined or not with antimicrobial proteins and other non-antimicrobial domains

There is a wide range of fusion partners (fusion tags) that have been used, as a strategy to properly express HDPs in a soluble form (Table 1). These solubility enhancing domains assist with correct folding and promote the expression to the soluble fraction of the protein of interest. In addition to enhance HDP solubility, tags might also protect against proteolysis, which HDPs are prone to due to their small size [82]. Some widely used solubility tags that tend to yield high levels of HDPs in the cytoplasm of *E. coli* are GST, Trx, GFP, SUMO and Silk (Table 1, Fig. 4A) [43, 83–88]. Although they need to be studied empirically on a case to case basis, in general they considerably improve the solubility of many recombinant proteins [89].

An interesting approach is the use of the N-terminal domain of spider silk as a fusion tag, which has been proven to yield up to 8 times more soluble protein compared to other frequently used expression tags and allows the expression of otherwise difficult to express peptides and proteins, such as the HDP precursor of LL-37 (hCAP18) [90]. Alternatively, acidic peptides can also be used, since they offer a charge-charge interaction that neutralizes the potential bactericidal effect of HDPs, avoiding the death of the host microorganism [91].

However, in many cases, there is a need to remove the carrier protein to isolate the peptide of interest. And often, removing the fusion partner requires expensive enzymatic cleavage or toxic reagents, such as cyanogen bromide or enterokinase hydrolysis [92, 93].

To overcome tag removal hurdles, self-cleaving tags (Fig. 4B), such as inteins can be used. Inteins are protein segments that can cut themselves from their precursors and re-join the flanking regions, also known as protein splicing [94]. This system has been used to express a cathelicidin, using *B. subtilis* as a host, allowing to purify the HDP by affinity chromatography and self-cleave in one step [95]. Self-cleaving tags, therefore, enable purification and cleavage in a single step, saving time, labor and reducing cost, but have an inherent risk of incomplete or uncontrolled cleavage [96].

### Multidomain HDPs

Some researchers have explored the use of fusion partners that add other functions that go beyond just helping to fold and express the recombinant HDPs. An example of this are cationic elastin-like polypeptides (ELP), that have successfully been used to purify a fusion hybrid of cecropin A and D without the need of chromatography [97]. ELP tags allow to form reversible spherical aggregates that allow to precipitate the fused HDPs under certain temperature conditions, in a process called inverse transition cycling, thus simplifying downstream processing, in addition to allowing HDP expression.

A different example, that shows how fusion partners might add new features to HDPs, is the broad-spectrum plant defensin HDP C6, which can be fused to a peptide pheromone (cCF10) to add specificity to the original HDP. The cCF10 pheromone domain is species-specific and binds to the bacterial membrane of *Enterococcus faecalis* with high affinity, an appealing addition to the original antimicrobial activity that allows for the precise killing of *E. faecalis* while avoiding potential off-target killing of beneficial microorganisms found in the host microbiome, which is what most the conventional antibiotics do [98]. This type of multidomain approach is also known as specifically targeted antimicrobial peptides (STAMPs) [99]. Many combinations of *killing* and *targeting* domains can be tried, where wild-type, rationally enhanced or artificial sequences can be used [100–103]. In all cases, these hybrid antimicrobials show improved antimicrobial activity, selectivity, and kinetics against their specific targets [87, 98]. Nonetheless, there still are inevitable bactericidal effects on other bacteria.

**Fig. 5:**
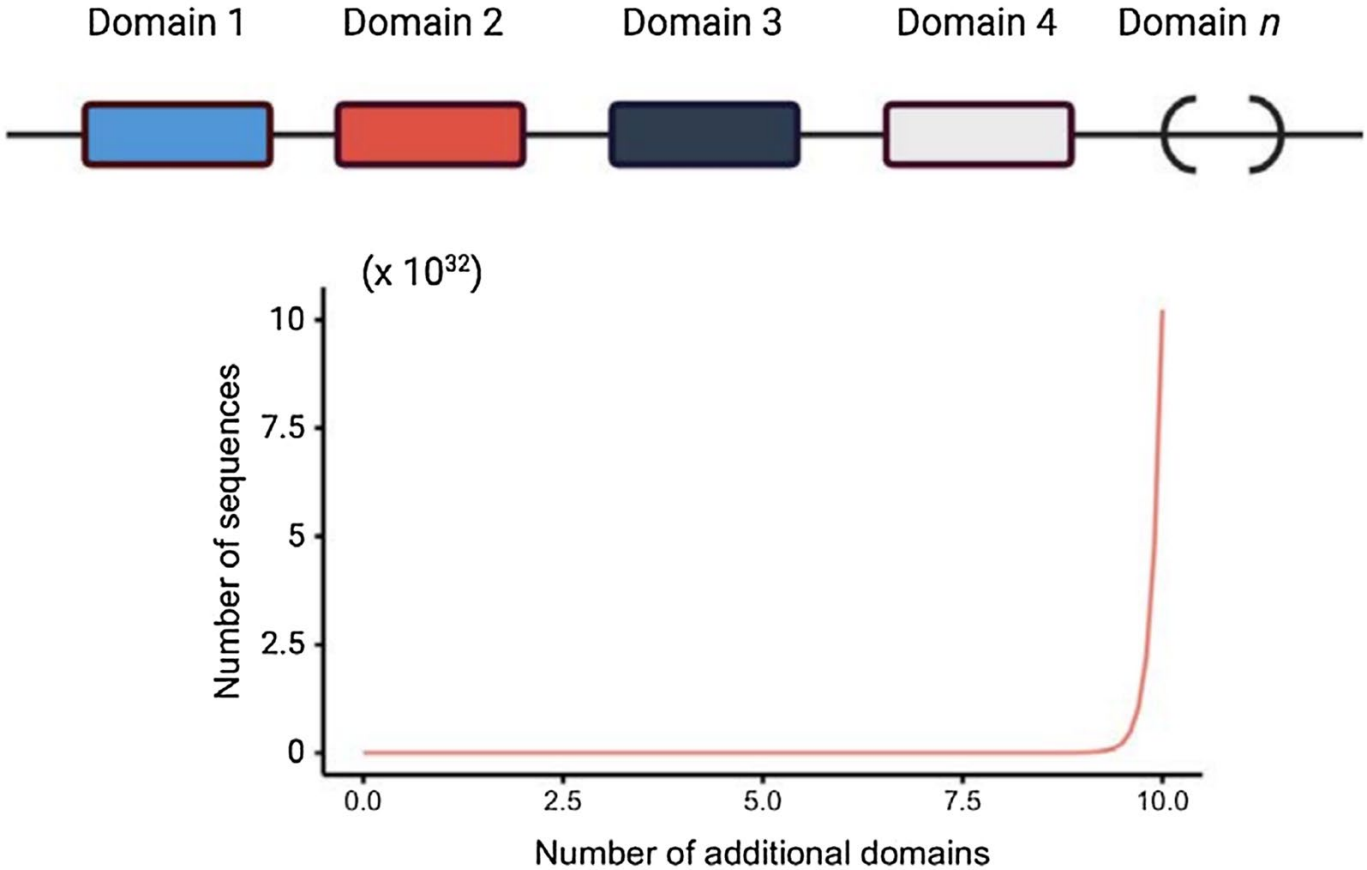
The boundless possibilities of multidomain HDPs. There is a vast range of domains to choose from for each domain we might want to add to our chimeric HDP constructs, each one adding new functionalities, that might not necessarily be related to host defense (i.e. target specificity)

The use of additional HDPs as fusion partners of other HDPs represents a unique strategy for the generation of recombinant multidomain HDPs (Fig. 5D), without the need for a carrier protein that needs to be removed downstream. For example, the pore-forming HD5 has been fused to an enzyme that hydrolyses bacterial membrane phospholipids, such as human XII-A secreted phospholipase A2 (sPLA_2_), generating a multidomain antimicrobial protein that can be successfully expressed recombinantly, and that attacks bacteria by using two completely different mechanisms [20]. This broad-spectrum multidomain construct, named JAMF1, was effective against several antibiotic resistant bacterial strains such as quinolone and carbapenem resistant *K. pneumoniae* (Fig. 5D). Similarly, in an effort to develop a vaccine against glyceraldehyde-3-phosphate dehydrogenase (GAPDH), an enzyme involved in the virulence of *Mycoplasma bovis*, different bovine HDPs were fused to GAPDH to boost the immune response against GAPDH (i.e. BMAP28, TAP and indolicidin) [104]. Interestingly, when HDPs are linked to enzymes, such as in the construct JAMF1 or GAPDH-HDP chimeras, both the enzyme and the HDP seem to keep the activities of the individual components, namely catalytic, antimicrobial, and immune-modulatory activities [21, 104].

#### Mutlidomain HDP fragment-stitching

Like the multidomain approach, segments (but not the full active sequence) of an HDP can be stitched together in a hybrid molecule that is a mix of the parental peptides, a process we named HDP *fragment-stitching*. Fragments of peptides such as the human cathelicidin LL-37, CM4 from a Chinese domestic silk moth and TP5, a fragment of the thymus hormone, have been used in one study to create hybrid antimicrobial that works against enterotoxic *E. coli* [105]. Fragment-stitching might be useful to remove undesired activities from a parental peptide, such as the hemolytic activity of LL-37, while generating new HDPs.

## Synergy of recombinant HDPs and antibiotics

The performance of antibiotics against some antimicrobial resistant (AMR) bacteria or in bacterial living resistant forms such as biofilms could be improved by their combination with other drugs. Numerous studies have assessed this principle by using synthetic HDPs [106] and antibiotics, demonstrating a clear synergy and beneficial effects on infection treatment. The main advantage of this strategy is to reduce the dose of each drug and consequently the possible toxic effects and eventually combine different mechanisms to control bacterial survival along with the emergence of bacterial resistances. In the context of recombinant HDPs, not many studies have been carried out. However, if synthetic HDPs can synergize with antibiotics, so should their recombinant version. To explore this, researchers tested the synergy of mouse β-defensin 3 (rMBD3) with different antibiotics against bacterial and yeast drug-resistant strains in vitro [107]. Interestingly, they found that the anti-methicillin-resistant *S. aureus* (MRSA) activity of rMBD3 in combination with ampicillin was synergistic, but it was not effective against methicillin sensible *S. aureus* [107]. Combinations of rMBD3 with itraconazole, amphotericin or 5-fluorocytosine were synergistic against two tested *Candida albicans* strains. These results support the potential of recombinant HDP to improve the activities of conventional antibiotics [107] and suggest that the same mechanism that makes bacteria resistant to a certain antibiotic might make them more vulnerable to combinatorial treatments.

## Conclusions

HDPs hold promise as new candidates to combat antibiotic-resistant pathogens. There is a clear need for new potent HDP candidates and the means to produce them efficiently and at a low-cost. Research on different expression systems will effectively accelerate our ability to increase production yields, peptide structure and bioactivity and provide access to engineered microbial cell factories that work universally well for most HDPs. But the optimal expression systems are only one part of the equation.

New recombinant HDP forms, such as IBs and encapsulated HDPs, are also worth considering. They offer properties that the soluble form does not provide, including higher stability, a slower release profile, reduced HDP toxicity or on-site activation. Besides, IBs should be exploited as both a new antimicrobial HDP format and as a treasure trove of bioactive, functional, and soluble HDPs.

Finally, strategies to optimize HDP production, such as fusion tags and multidomain HDPs, open the door to designing newly added functionalities, such as the precise killing of a pathogen, while avoiding off-target effects on probiotic bacteria. Moreover, a well-crafted tag strategy not only increases the uses of a recombinant HDP but also should allow for its production without the need of downstream tag removal. Collectively, finely-tuned recombinant approaches show promise for much-needed sustainable, inexpensive, and larger-scale production of antimicrobial peptides as well as other peptide therapeutics and in turn, allow these molecules to enter clinical practice.

## Data Availability

The datasets supporting the review are referenced throughout the article.

## Abbreviations

ARB: Antimicrobial resistant bacteria
AMP: Antimicrobial peptide
HDP: Host-defense peptide
SPPS: Solid-phase peptide synthesis
PTMS: Post-translational modifications
GRAS: Generally Recognized as Safe
CHO: Chinese Hamster Ovary
HSA: Human serum albumin
AMR: Antimicrobial resistance
rMBD: Recombinant mouse beta defensin
MRSA: Methicillin resistant *S. aureus*
IB: Inclusion bodies
HD5: Human alpha defensin 5
CAP: Cysteine rich acidic peptide
MIC: Minimal inhibitory concentration
PLGA: Poly(lactic-co-glycolic acid)
NAC: *N*-Acetyl cysteine
PEG: Polyethylene glycol
hCAP: Human cathelicidin antimicrobial protein
ELP: Elastin-like polypeptides
cCF10: Cell-associated pheromone peptide
STAMPS: Specifically targeted antimicrobial peptides
sPLA_2_: Secreted phospholipase A_2_
GAPDH: Glyceraldehyde 3-phosphate dehydrogenase
